# Simultaneous determination of nereistoxin insecticides in foods of animal origins by combining pH-dependent reversible partitioning with hydrophilic interaction chromatography-mass spectrometry

**DOI:** 10.1038/s41598-022-14520-3

**Published:** 2022-06-17

**Authors:** Seung-Hyun Yang, Hoon Choi

**Affiliations:** 1 Department of Life and Environmental Sciences, Wonkwang University, Iksan, 54538 Republic of Korea; 2 Institute of Life Science and Natural Resources, Wonkwang University, Iksan, 54538 Republic of Korea

**Keywords:** Mass spectrometry, Environmental chemistry

## Abstract

Although nereistoxin insecticides (NIs) are banned for animal husbandry operations, they are still used because of their high insecticidal activities. Therefore, a reliable residue analysis method for the simultaneous detection of cartap, bensultap, thiocyclam, and nereistoxin in foods of animal origins, including beef, pork, chicken, milk, and eggs, was developed using hydrophilic interaction liquid chromatography–mass spectrometry (HILIC–LC–MS/MS). The NIs were extracted with an acidic cysteine and formate buffer solution and hydrolyzed to nereistoxin. The molarity and pH of the buffer were optimized at 20 mM and 3, respectively, to keep the pH of the extracts at 4–5. pH-dependent acid–base partitioning coupled with salting-out-assisted liquid–liquid extraction using acetonitrile was performed for purification and for the direct introduction of the extracts to LC. The optimal pH values were 5 and 9 for the acid–base partitioning. Nereistoxin quantitation was achieved with consistent column retention (RSD < 0.6%) and a high degree of separation (*N* > 10^6^). The matrix-dependent method limit of quantitation was 2 μg nereistoxin/kg, and the calibration curve showed good linearity (R^2^ > 0.998). The recovery efficiencies were in the range of 89.2–109.9% with relative standard deviations less than 10%, and matrix effects did not exceed ± 10%, which satisfied the criteria outlined in the European SANTE/12682/2019 guidelines.

## Introduction

Pesticides and veterinary drugs are indispensable in agriculture and animal husbandry operations, respectively. Pesticides have been widely used to control various insects, diseases, and weeds in the agricultural industry^1^. In animal husbandry, veterinary drugs are used to manage insect pests and animal diseases, which helps increase production efficiency and improve the quality of livestock and poultry products^2^. However, the intake of foods and drinks containing pesticides or veterinary drugs can have harmful effects on consumers depending on the residue levels, intake amounts, and toxicity to living organisms. Therefore, government agencies in several countries have established safety standards such as the maximum residue limits (MRLs) for food production.

From the viewpoint of husbandry, where the primary goal is maximum production, intensive farming represents the best industrial management system. However, it also increases the number of insect pests and diseases, leading to the heavy use of veterinary drugs during animal husbandry. Husbandry operations employ many different types of veterinary drugs, including organophosphates, carbamates, and pyrethroids, to control poultry red mite infestations and prevent economic losses due to reduced egg production^3,4^. In 2007, European authorities reported that eggs and chickens were contaminated with pesticides banned in livestock farming, such as fipronil. Eggs tainted with insecticides have also been discovered in Korea, which has resulted in major food safety-related issues. Because few veterinary drugs are registered as insecticides in Korea and resistance to approved acaricides is prevalent^4,5^, insecticides are often illegally used for cage-grown animals.

Cartap, bensultap, and thiocyclam belong to the nereistoxin insecticides (NIs) class, which are metabolized to nereistoxin and bind to nicotinic acetylcholine receptors, thereby causing paralysis in the central nervous system of insects^6,7^. These nereistoxin analogs have been widely applied to chewing and sucking insects, such as Lepidoptera and Coleoptera in cereals and fruits, *Chilo suppressalis* and *Cnaphalocrocis medinalis* in rice, and *Leptinotarsa decemlineata* and *Plutella xylostella* in potatoes, cabbage, and other vegetables^7^. Owing to their high insecticidal activity against a wide range of insects, NIs are illegally used to control insects, such as poultry red mites, in livestock and poultry farms in Korea. Therefore, an analytical method for NIs is required to determine the presence of trace levels of unapproved pesticides in foods of animal origins.

Many analytical methods for determining NIs levels in biological fluids, water, sediments, soils, and foods of plant origins have been developed. Several sample preparation techniques, including liquid–liquid extraction (LLE), solid phase extraction (SPE), solid phase microextraction (SPME), and dispersive SPE (d-SPE), have been successfully employed for purification. Colorimetric assays containing gold nanoparticles^6,8^ and instrumental methods, such as gas chromatography–flame photometric detector (GC–FPD)^9,10^, gas chromatography–mass spectrometry (GC–MS)^11–13^, and liquid chromatography–mass spectrometry (LC–MS)^14–16^, have been utilized for the identification and detection of NIs. However, these previously developed analytical techniques have various limitations, including poor reproducibility due to the high volatility of nereistoxin during the evaporation process^9–11^ and poor recovery rates of nereistoxin analogs, such as thiosultap, during simultaneous analyses^15^. Recently, the QuEChERS (Quick, Easy, Cheap, Effective, Rugged, and Safe) extraction method has been utilized for sample pretreatment using various d-SPE materials, including Ti_2_C nanosheets, organic polymers, amorphous carbon nanoparticles, and covalent organic frameworks^17–20^. Cartap, thiocyclam and nereistoxin could be extracted using acetonitrile or ethyl acetate, but bensultap could not be extracted, indicating that it is difficult to apply the QuEChERS method for simultaneous analysis. In summary, several analytical methods have been reported, but they have not shown good extraction and recovery efficiencies for all NIs, including thiosultap and bensultap.

To the best of our knowledge, no analytical methods for the simultaneous quantification of NIs and their metabolite nereistoxin in foods of animal and plant origins have been established previously. Hence, this study aims to develop a reliable residue analysis technique for the determination of trace levels of cartap, bensultap, thiocyclam, and nereistoxin in foods of animal origins, which could potentially be utilized as an official method for compliance purposes. Because the definition for residues in foods of plant origins includes the sum of cartap, bensultap, thiocyclam, and nereistoxin (expressed as nereistoxin), a “common moiety” method involving the hydrolysis of NIs to nereistoxin is proposed in the present study. The corresponding extraction, hydrolysis, purification, and instrumental analysis procedures are optimized according to the European SANTE/12682/2019 guidelines.

## Materials and methods

### Reagents and materials

Analytical standards of cartap (purity: 98.6%), bensultap (purity: 98.6%), and thiocyclam (purity: 93.7%) were purchased from Sigma–Aldrich (St Louis, MO, USA), and its metabolite nereistoxin (1000 ppm, 1 mL) was procured from Kemidas (Suwon, Republic of Korea). High-performance liquid chromatography (HPLC)-grade acetonitrile was obtained from J.T. Baker (Avantor, Radnor, PA, USA); *n*-hexane and ammonium hydroxide (purity: 25–30%) were purchased from Daejung Chemicals & Materials (Siheung, Republic of Korea); and formic acid (purity: 99%) was acquired from FUJIFILM Wako Pure Chemical Corporation (Osaka, Japan). Ten normal sulfuric acid and sodium hydroxide solutions were obtained from Merck (Darmstadt, Germany) and Biosesang (Seongnam, Republic of Korea), respectively. Anhydrous L-cysteine hydrochloride (> 98%) and nickel(II) chloride (> 98%) were purchased from Sigma–Aldrich (St Louis, MO, USA). Sodium chloride and ammonium formate (> 97%) were obtained from Junsei Chemical Co., Ltd. (Tokyo, Japan) and Daejung Chemicals & Materials (Siheung, Republic of Korea), respectively. Deionized water (18.2 MΩ cm) was prepared using a Direct-Q®3 UV water purification system (Millipore, Bedford, MA, USA).

### Preparation of standard solutions

Stock solutions of NIs and nereistoxin were prepared by weighing their reference standards and dissolving them in acetonitrile at concentrations of 100 μg/mL. Individual working standard solutions was prepared from each stock solution and diluted with acetonitrile to final concentrations of 1–3 μg/mL for fortification experiments. The stock and working standard solutions were stored at − 20 °C in a freezer. A series of working solutions for the nereistoxin standard (100 μL) were added to blank sample extracts (400 μL) to obtain matrix-matched standard solutions with concentrations of 0.1–20 μg/L.

### Sample preparation

Foods of animal origins, including beef (sirloin), pork (pork belly), chicken (leg meat), milk, and eggs, were obtained from local markets that sell organic products. The meat samples were ground with an MG516 Pro 1600 meat grinder (Kenwood, Havant, UK), while the milk and eggs were homogenized using a T25 Digital Ultra-Turrax® homogenizer (IKA-Werke GmbH & Co., Staufen, Germany). The homogenized samples (meat: 10 g, milk: 10 mL, eggs: 10 g) without pesticides were fortified with the individual working standard solutions. The spiked samples were incubated for 20 min before extraction.

### LC-triple quadrupole mass spectrometry parameters for nereistoxin analysis

Nereistoxin was analyzed using a Shimadzu Nexera HPLC system coupled to a Shimadzu LCMS-8045 triple quadrupole mass spectrometer with an electrospray ionization (ESI) source (Shimadzu Corp., Kyoto, Japan). Three different analytical columns, including XBridge C18 (100 mm × 2.1 mm, i.d. 3.5 μm; Waters, Dublin, Ireland), XBridge hydrophilic interaction liquid chromatography (HILIC; 150 mm × 2.1 mm, i.d. 3.5 μm; Waters, Dublin, Ireland), and XBridge Amide (150 mm × 2.1 mm, i.d. 3.5 μm; Waters, Dublin, Ireland) were investigated at a flow rate of 400 μL/min using a water/acetonitrile (10:90, v/v) mobile phase for chromatographic performance optimization. In addition, the separation effects observed at different elution orders and with the addition of 0.1% (v/v) formic acid (serving as a modifier) were examined. The column temperature was 40 °C, and the injection volume was 5 μL.

The source parameters for MS analysis optimized in the positive ion mode included an interface voltage of 4.0 kV, interface current of 19.8 μA, detector voltage of 1.94 kV, interface temperature of 250 °C, desolvation line temperature of 250 °C, heat block temperature of 300 °C, nebulizing gas flow of 3 L/min, drying gas flow of 10 L/min, and heating gas flow of 10 L/min. The nebulizing and drying gases contained nitrogen produced by a nitrogen generator (Euroscience, Seongnam, Republic of Korea). The collision gas consisted of argon (purity: 99.999%) maintained at a pressure of 17 kPa. When nereistoxin was used as the target compound, the precursor ion mass at a Q1 prebias voltage of 28 V was *m/z* 149.75, and the masses of the transition ions selected for quantification and identification at collision energies of 15 and 23 V were equal to *m/z* 105.05 and 71.00, respectively. A Shimadzu LabSolutions software (ver. 5.97) was used for instrument control, data acquisition, and data processing.

### Retention characteristics and column efficiency

To determine the HPLC retention characteristics of nereistoxin in various sample media, a capacity factor (*k*) was calculated from the retention time and adjusted retention time (t_R_'). The elution time of uracil was used as the dead time. In addition, to estimate the HPLC column efficiency, the number of theoretical plates (*N*) was computed from the retention time and peak width, while the height of these plates (*H*) was calculated from *N* and the column length^21^.

1 $$k = \, t_{R}\,^{\prime}/t_{0} \;\;\;\;\;\;\;\;\;\;\;\;t_{R}\,^{\prime}:{\text{adjusted retention time of nereistoxin}};t_{0} :{\text{dead time}}$$

2 $$t_{R} \, ^{\prime} \, = \, t_{R} - \, t_{0} \;\;\;\;\;\;\;\;\;\;\;\; t_{R} :{\text{retention time of nereistoxin}}$$

3 $$N = \, 5.545 \, \times \, (t_{R} /W_{h} )^{2} \;\;\;\;\;\;\;\;\;\;\;\; W_{h} :{\text{peak width at}}\;\raise.5ex\hbox{$\scriptstyle 1$}\kern-.1em/ \kern-.15em\lower.25ex\hbox{$\scriptstyle 2$} \;{\text{height}}$$

4 $$H = {\text{ Column length }}\left( {{\text{mm}}} \right)/N$$

### Extraction of NIs and their decomposition to nereistoxin

Ten grams or milliliters of the homogenized samples containing beef, pork, chicken, milk, and eggs was weighed into a 50-mL PTFE centrifuge tube and mixed with 40 mL (meat) or 30 mL (milk and eggs) of an acidic buffer solution consisting of 2% cysteine and 20 mM ammonium formate (pH 3, adjusted with ammonium hydroxide) as the extraction solution. The tube was vortexed for 5 min at 1500 rpm with a shaker (2010 Geno/Grinder®, SPEX® SamplePrep, Metuchen, NJ, USA) to extract the NIs and nereistoxin and then centrifuged for 10 min at 4000 rpm using a centrifuge (Combi-406, Hanil Scientific Inc., Gimpo, Republic of Korea) to obtain the supernatant. Note that the supernatant obtained for milk and eggs was diluted to 40 mL with the extraction solution. For decomposition to nereistoxin, the supernatant (20 mL) was transferred to another 50-mL centrifuge tube containing 1.5 mL of 3% nickel chloride and 1.5 mL of ammonium hydroxide. The mixture was shaken vigorously by hand, stored for 30 min at 70 °C, and cooled to room temperature.

### pH-dependent reversible acid–base partitioning with salting-out method

After decomposition, the hydrolysate was adjusted to pH 5 using a 10 N solution of sulfuric acid followed by the addition of 10 mL *n*-hexane to remove any interferences. The mixture was then vigorously shaken by hand for 1 min and centrifuged at 4000 rpm for 10 min. After discarding the organic supernatant (upper layer), the aqueous layer was carefully adjusted to pH 9 with a 10 N sodium hydroxide solution. Subsequently, 6 g of sodium chloride and 20 mL of acetonitrile were added to the mixture, and it was then vortexed and centrifuged at 4000 rpm for 10 min. The organic supernatant (upper layer) was collected and filtered through a 0.45-μm PTFE syringe filter before HPLC–triple quadrupole mass spectrometry (MS/MS) analysis.

### Matrix effects on nereistoxin quantitation

Matrix effects (MEs) were determined using the following equation: ME (%) = (S_matrix_ − S_solvent_)/S_solvent_ × 100, where S_matrix_ and S_solvent_ are the slopes of the calibration curves obtained using the matrix-matched standard solutions and working solutions in acetonitrile, respectively. At |ME|< 20%, the ME was weak or could be ignored; if |ME| varied from 20 to 50%, it was moderate; and at |ME|> 50%, the ME was considered strong^22,23^.

### Method validation

To validate the developed analytical method, the selectivity, limits of detection (LODs), limits of quantitation (LOQs), linearities, and recovery efficiencies were estimated for the studied compounds. Selectivity was determined by calculating the relative standard deviation (% RSD) of the retention times and the intensity ratios of the two transition ions obtained for the matrix-matched calibration solutions and blank sample extracts. The instrumental LOD (ILOD) and LOQ (ILOQ) of nereistoxin were computed by analyzing aliquots of nereistoxin matrix-matched standard solutions with concentrations ranging from 0.01 to 1 μg/L. ILOD and ILOQ were defined as the concentrations with signal-to-noise (S/N) ratios of the quantifier ion intensities equal to 3 and 10, respectively^24^. The matrix-dependent LOQs (MLOQs) of the NIs were defined as the lowest concentration determined from the ILOQ of nereistoxin, injection volume, dilution rate, and nereistoxin conversion factor in the analytical method^25^. The nereistoxin conversion factors were calculated by dividing the molecular weight of nereistoxin (M.W. 149.3) by the molecular weight of cartap (M.W. 237.3), bensultap, (M.W. 431.6), and thiocyclam (M.W. 181.3), respectively. The conversion factors were 0.6 for cartap, 0.3 for bensultap, and 0.8 for thiocyclam. A series of matrix-matched standard solutions with nereistoxin concentrations of 0.3–20 μg/L in foods of animal origins were prepared for HPLC–MS/MS analysis. The linearity of the calibration curve was evaluated by calculating the coefficient of determination (R^2^) in regression analysis using SPSS software (ver. 18.0, SPSS Inc., Armonk, NY, USA). Instrumental repeatability was verified by calculating the % RSD of the peak area after seven consecutive analyses of the matrix-matched standard solution with a nereistoxin concentration of 10 μg/L. Recovery efficiency tests were conducted to determine the accuracy and precision of the developed method. The control samples were fortified at three different levels (MLOQ, 10 MLOQ, and 50 MLOQ) by spiking them with appropriate amounts of the working solutions of the NIs and nereistoxin. All analyses were repeated five times. The mean recoveries and % RSD values were calculated to determine method accuracy and precision, respectively. The greenness of the developed method was evaluated by the Analytical Eco-Scale method to confirm its environmental impact^26^. The score on the Eco-Scale was determined by subtracting the penalty points from the ideal value of 100.

## Results and discussion

### Optimization of MS/MS parameters

The MS/MS parameters were optimized only for nereistoxin because the NIs including cartap, bensultap, and thiocyclam, were converted to nereistoxin during sample preparation. A multiple reaction monitoring (MRM) mode was used to perform compound quantification and identification. First, the intensity of an abundant protonated ion [M + H]^+^ (*m*/*z* 149.75) selected as the precursor ion was recorded for nereistoxin (molecular mass: 149.03) in full-scan mode during the electrospray ionization process. Subsequently, the collision energy was gradually increased from 5 to 35 V to obtain fragment ions with higher intensities from the precursor ion. Two peaks with high *m/z* ratios (105.05 and 71.00) and abundances were present in the fragment ion spectrum; therefore, they were utilized as the monitoring ions for quantification and identification purposes, respectively. The ion ratio of the two MRM transitions was 8.3%.

### Optimization of the column and mobile phases

**Figure 1:**
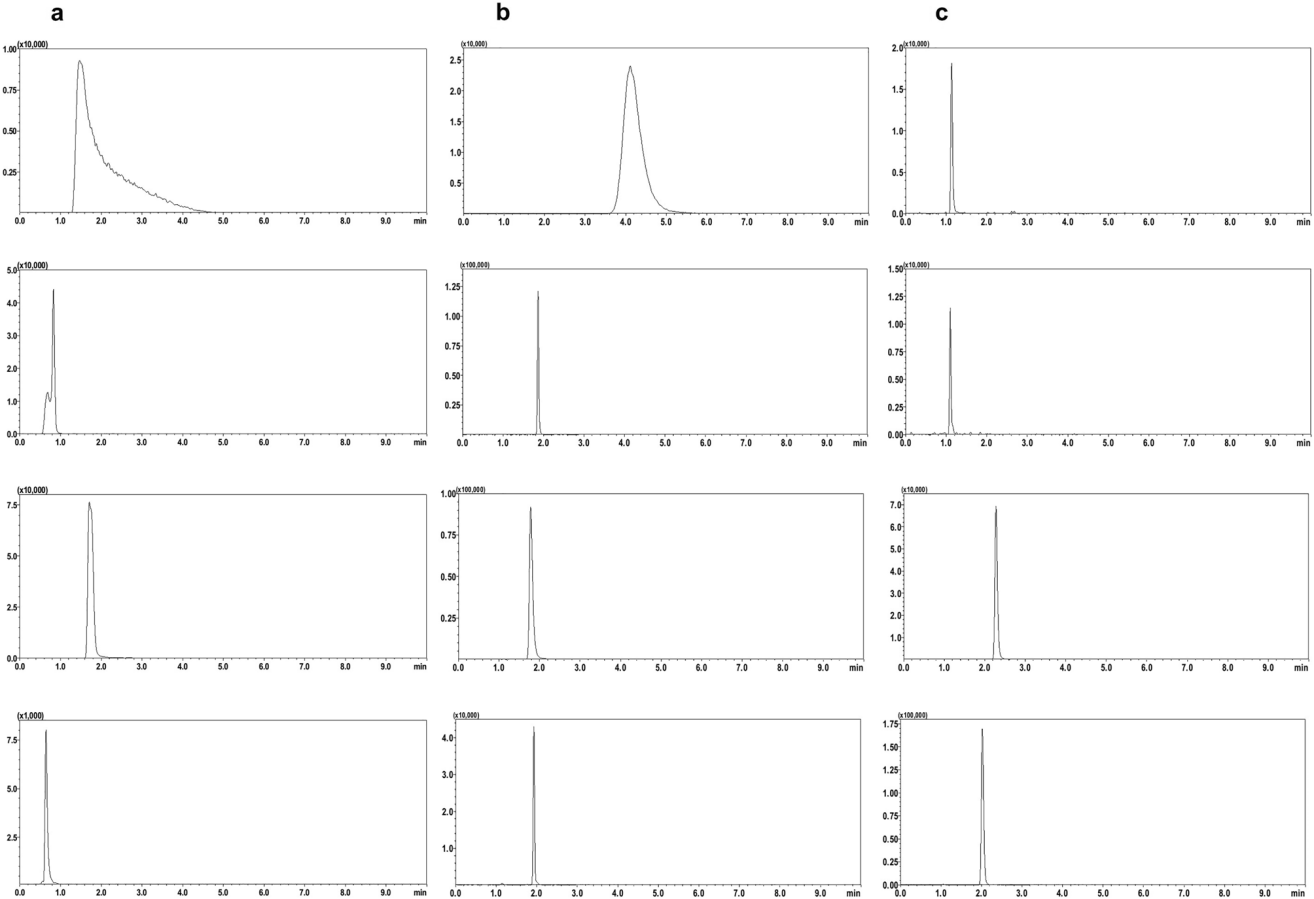
LC–MS/MS chromatograms of the standard solution, matrix-matched solution, standard solution with a mobile phase containing 0.1% formic acid, and matrix-matched solution with a mobile phase containing 0.1% formic acid at 20 ng nereistoxin/mL with C18 (**a**), HILIC (**b**), and Amide (**c**) columns.

Chromatographic parameters were determined to evaluate the effects of different columns and mobile phase compositions on nereistoxin separation and to achieve maximum nereistoxin sensitivity and resolution. The XBridge C18 column used commonly in HPLC analysis was tested. Because nereistoxin is a polar compound, a hydrophilic interaction liquid chromatography (HILIC) column was also tested, which is a newly developed normal phase column that promotes the retention and separation of extremely polar compounds^27^. HILIC columns have been recommended for mobile phases containing 5–50% water by the manufacturer. These columns were compared using a mobile phase consisting of water/acetonitrile (A:B = 10:90, v/v). For the XBridge C18 column, the nereistoxin peak shape was improved by adding 0.1% formic acid to the mobile phase; however, nereistoxin was not retained in the column at any level of formic acid addition (Fig. 1a). In brief, the acidic mobile phase enhanced the nereistoxin peak shape because the molecule existed in the single ionized form under acidic conditions. Meanwhile, nereistoxin was more strongly retained in the XBridge HILIC column than in the silica-based reversed-phase column (Fig. 1b). Moreover, sharper nereistoxin chromatographic peaks were obtained for sample extracts after a 16-fold increase in the number of theoretical plates (Table 1, Fig. 1b). *N* is an index of column efficiency. The larger the *N* value is, the sharper the peaks and the higher the separation quality. However, because of poor reproducibility, relatively large fluctuations in the retention time were observed during the consecutive analyses of the sample extracts. The XBridge Amide column was examined instead of the XBridge HILIC column, which is an HILIC column with a trifunctional amide ligand, enabling the retention and separation of polar compounds over a wide pH range of pH 2–11. A sharp nereistoxin peak was also detected for the sample extracts analyzed using the XBridge Amide column. The nereistoxin retention time and the *N* value were increased by adding 0.1% formic acid to the mobile phase (Table 1, Fig. 1c). As a result, nereistoxin eluted consistently at a retention time of 2.028 min, W_h_ of 0.015 min, and *N* of > 10^6^. This analytical method exhibited satisfactory chromatographic performance in terms of sensitivity, selectivity, resolution, and retention. Thus, these optimized analytical conditions were subsequently used in the pretreatment procedures.

**Table 1:** HPLC chromatographic characteristics of nereistoxin in a mobile phase consisting of water/acetonitrile (10:90, v/v) with 0.1% formic acid. t_R_–retention time; t_0_–dead time; t_R_'–adjusted retention time; *k*–capacity factor; *N*–number of theoretical plates; *H*–height of theoretical plates.

Column	Solvent	t_R_(min)	t_0_(min)	t_R_'(min)	*k*	*N*	*H*(mm)
C18	Acetonitrile	1.708	0.626	1.082	1.728	7981	0.013
	Beef extract	0.643	0.626	0.017	0.027	3142	0.032
HILIC	Acetonitrile	1.781	1.299	0.482	0.371	13,559	0.011
	Beef extract	1.929	1.299	0.630	0.485	51,536	0.003
Amide	Acetonitrile	2.132	0.886	1.246	1.406	26,203	0.006
	Beef extract	2.028	0.886	1.142	1.289	101,265	0.001

### Extraction with acidic buffer and decomposition to nereistoxin under alkaline conditions

**Figure 2:**
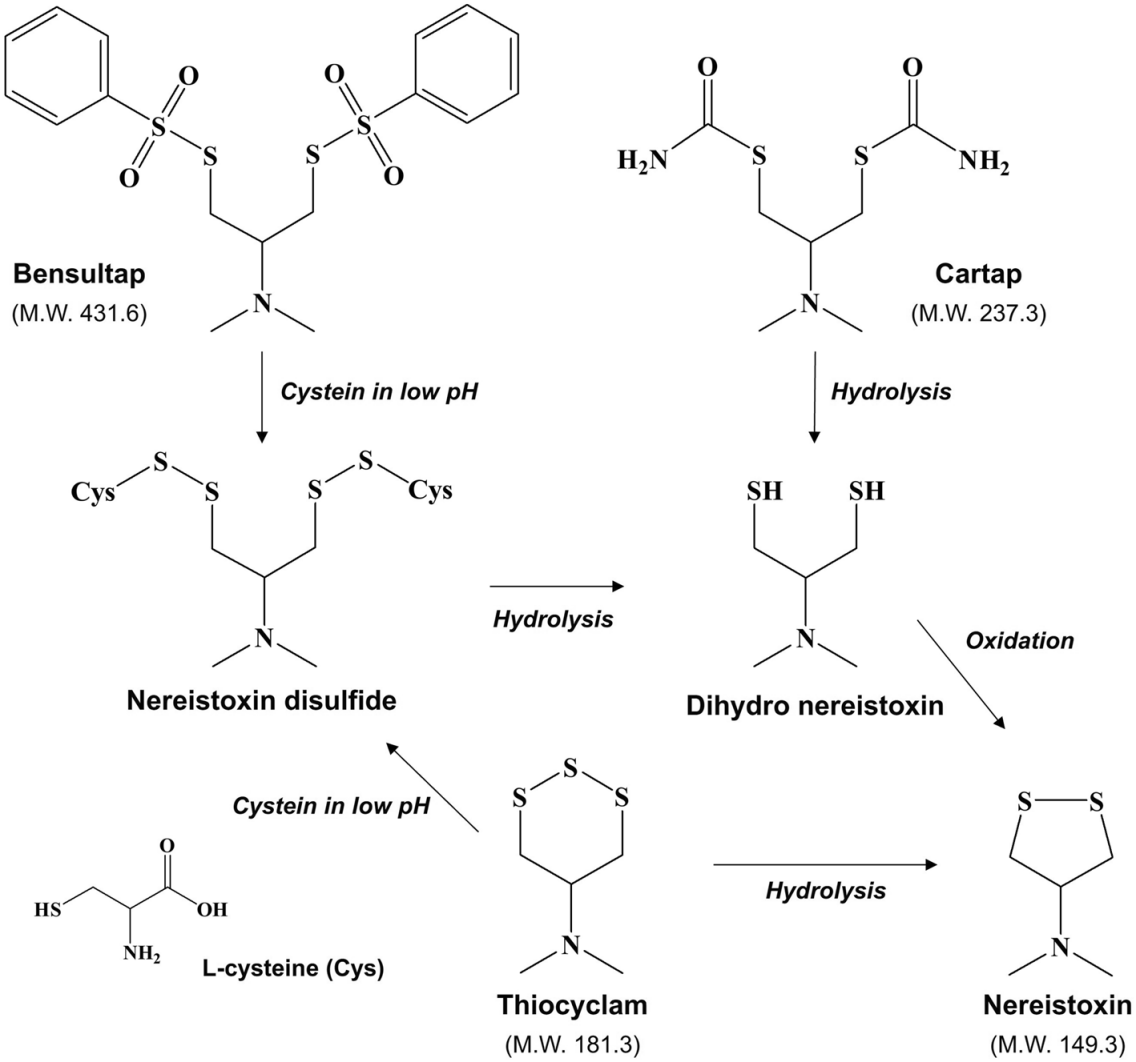
Conversion pathways of the nereistoxin insecticides during the pretreatment procedure.

Cartap is a polar and water-soluble compound that is stable under low pH conditions^14^. It decomposes to nereistoxin through a dihydro-nereistoxin intermediate by hydrolysis and oxidation under neutral or alkaline conditions^6,11,28^(Fig. 2). Bensultap is moderately polar (log K_OW_ 2.28) and stable at pH < 5. It is poorly extracted by acetonitrile and is easily hydrolyzed to nereistoxin monoxide under aqueous conditions^7,9^; therefore, the QuEChERS method is difficult to apply. Inoue & Yamamoto suggested converting bensultap to nereistoxin disulfide in an acidic cysteine solution for higher extraction efficiency^9^ because of its incomplete hydrolysis to nereistoxin monoxide and its partial absorption by the sample (Fig. 2). Thiocyclam is polar (log K_OW_ -0.07 for thiocyclam hydrogen oxalate) and can be extracted with acetonitrile under acidic conditions. Previous studies have shown that it can be hydrolyzed in water and biological media and then transformed to nereistoxin through a reaction with cysteine^11,15^(Fig. 2). Nereistoxin, a metabolite of cartap, bensultap, and thiocyclam, is a polar and basic compound that is more stable than the parent compounds. As shown in Fig. 2, NIs and nereistoxin are soluble in water, while bensultap and thiocyclam can be converted to nereistoxin disulfide in the presence of excess cysteine under low pH conditions. Considering the previously obtained data, a 2% cysteine solution in 0.02 N HCl with pH 4 was selected as the extraction medium for all NIs and nereistoxin.

The decomposition of nereistoxin disulfide to nereistoxin was carried out by a method developed by Inoue & Yamamoto^9^, which is similar to the official methods utilized by the Japanese and Korean authorities^29,30^. Nereistoxin disulfide was easily decomposed to nereistoxin by basifying the mixture with ammonium hydroxide in the presence of excess cysteine (Fig. 2)*.* Cysteine is water-soluble and exists in a zwitterionic form at pH 4; however, it precipitates as cystine under basic conditions^31^. Cystine can be separated and removed from the supernatant via the following partitioning process. A nereistoxin polymer can be partially formed from dihydro-nereistoxin in the presence of excess cysteine. Therefore, nickel chloride was added to chelate dihydro-nereistoxin during the decomposition step, which suppressed the formation of the nereistoxin polymer but did not affect nereistoxin formation^9^. The formation of nereistoxin disulfide in the 2% cysteine solution at pH 4 and its decomposition to nereistoxin by alkaline hydrolysis were studied for each standard solution (1 ppm, 1 mL). Cartap, bensultap, and thiocyclam were fully decomposed to nereistoxin and recovered with an efficiency of more than 95%.

**Figure 3:**
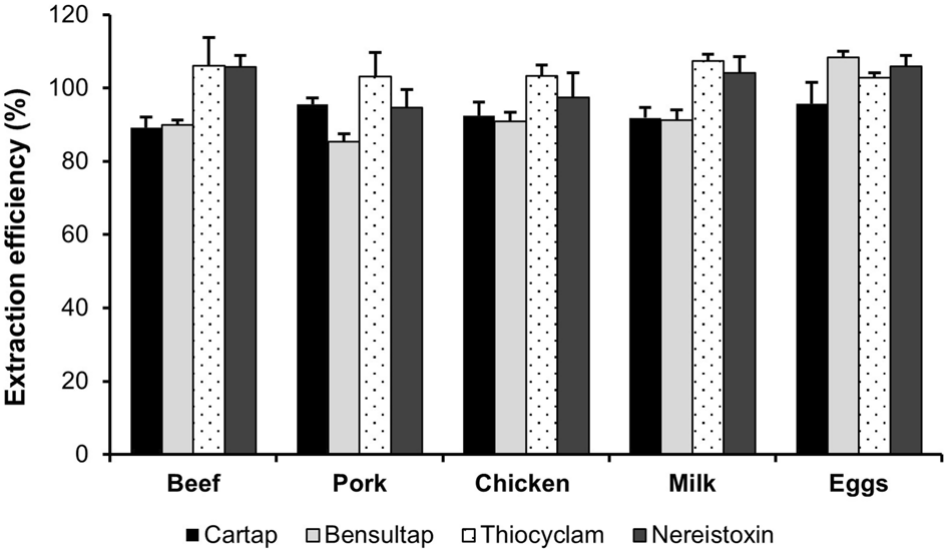
Extraction efficiencies of the nereistoxin insecticides and nereistoxin determined for an extraction solution containing 2% cysteine and 20 mM ammonium formate at pH 3.

The pH of the extracts strongly affected the extraction efficiency and formation of nereistoxin disulfide. As shown in Table 2, the pH values of the milk and egg samples varied from 6.2 to 7.7. Although the pH of the extraction solvent was adjusted with hydrogen chloride, it was difficult for the extracts of all foods of animal origins to attain a pH value of 4–5. Thus, the effects of pH and the molarity of the buffer solution on the pH of the extracts were investigated by varying the buffer pH from 3 to 4 and by varying the molarity from 5 to 20 mM. Formate buffer was selected as the extraction solvent because formic acid was used as the mobile phase in the LC–MS/MS experiments. The mixture of 2% cysteine (pK_a_ 1.77) and ammonium formate had a pH below 2, and the pH of the buffer was adjusted with ammonium hydroxide. An extraction solvent with pH 3 containing 2% cysteine and 20 mM ammonium formate resulted in the optimal pH values in the extracts (Table 2). The obtained extraction efficiencies of NIs and nereistoxin were in the range of 85.4–108.4% (Fig. 3). Thus, an aqueous solution of 2% cysteine and 20 mM ammonium formate adjusted to pH 3 with ammonium hydroxide was further used as the extraction solvent for foods of animal origins. During extraction, no lipid coagulation was detected in the meat samples, but protein coagulation was observed in the milk and egg samples; hence, their products were effectively removed through centrifugation. The volume of extraction solvent for the liquid samples, such as the milk and egg samples, was adjusted to 30 mL, by taking into account the total volume of the mixture of the sample and solvent.

**Table 2:** Effects of various extraction solvents on the pH values of the livestock product extracts.

Extraction solvents	pH of the extract				
	Beef	Pork	Chicken	Milk	Eggs
Before addition of solvents	–	–	–	6.2–6.4	6.8–7.7
2% cysteine, 0.02 N HCl pH 3	4.9–5.2	5.5–5.6	5.6–5.7	5.4–5.7	6.0–6.1
2% cysteine, 5 mM HCOONH_4_, pH 3	4.6–4.8	4.7–4.8	4.8–4.9	4.6–4.7	5.5–5.6
2% cysteine, 5 mM HCOONH_4_, pH 4	5.0–5.1	6.0–6.1	5.6–5.7	5.0–5.9	6.0–6.1
2% cysteine, 10 mM HCOONH_4_, pH 3	4.4–4.5	4.2–4.4	4.7–4.8	4.2–4.6	5.1–5.2
2% cysteine, 10 mM HCOONH_4_, pH 4	5.2–5.3	6.1–6.3	5.6–5.8	6.1–6.2	6.4–6.5
2% cysteine, 20 mM HCOONH_4_, pH 3	4.3–4.5	4.2–4.3	4.5–4.6	4.0–4.3	4.7–4.8
2% cysteine, 20 mM HCOONH_4_, pH 4	5.3–5.4	6.5–6.6	5.8–5.9	6.4–6.5	6.5–6.6

### pH-dependent reversible acid*–*base partitioning and salting-out assisted liquid*–*liquid extraction

After decomposition, the hydrolysate contained various interfering polar compounds, including proteins, organic acids, and salts, because of the water extraction and salt addition, and it contained nonpolar compounds, such as lipids and fats, originating from the foods of animal origins. A pH-dependent reversible acid–base partitioning procedure was performed to remove these polar and nonpolar interferences^32^. This was possible because nereistoxin is a basic compound and can be converted to a dissociated or undissociated form through pH adjustment. Under acidic conditions, the dissociated form of nereistoxin is dominant, and it easily dissolves in aqueous solutions. On the other hand, nonpolar interferences were removed by extraction with organic solvents, such as *n*-hexane. Subsequently, the dissociated polar analyte in an aqueous solution was neutralized to the undissociated form by the addition of a base, such as sodium hydroxide. This form easily partitioned into the organic phase, thereby separating from the polar interferences.

In previous studies^9–11,29,30^, ethyl acetate and dichloromethane have been used as organic solvents for the liquid–liquid extraction of nereistoxin. Because nereistoxin is highly volatile, it can be easily lost during ethyl acetate or dichloromethane evaporation after liquid–liquid extraction. Recently, solid-phase microextraction (SPME) and dispersive liquid–liquid microextraction (DLLME) have been developed for the extraction and preconcentration of pesticides using small amounts of solvents. DLLME is a sample preparation technique using a water-miscible solvent as the dispersive solvent. Various kinds of dispersive solvents, such as chloroform, choline chloride:pivalic acid, hexane:ethyl acetate, and butanol, have been developed for pesticide residue analysis; however, they have all been associated with GC–ECD, GC–MS, and UV–VIS spectrophotometry^33–38^. Moreover, a SPME technique has also been developed for GC analysis^37^. Therefore, to introduce the extracts into the LC–MS/MS instrument without evaporation, a salting-out-assisted liquid–liquid extraction (SALLE) procedure using acetonitrile as the extraction solvent was conducted in this study. Acetonitrile is a water-miscible organic solvent that can be separated from the aqueous phase by the addition of excessive quantities of salts^39^. The addition of salts, such as sodium chloride, generally increases the ionic strength and decreases the analyte solubility in aqueous solutions^40^, which promotes analyte transfer into the organic layer. Moreover, the inorganic salts added in the extraction and decomposition step can be removed through the SALLE process due to their low solubility in acetonitrile.

**Figure 4:**
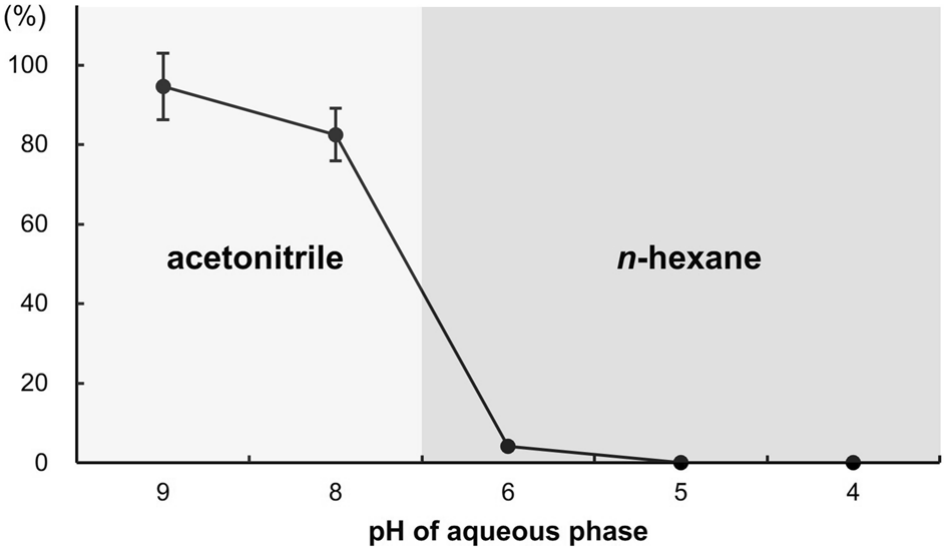
Partition efficiencies of nereistoxin with acetonitrile or *n*-hexane at various pH values.

Because the pK_a_ value of nereistoxin has not been accurately determined, the optimal pH value was obtained for the reversible acid–base partitioning. The pH of the aqueous phase was adjusted with 10 N sulfuric acid and 10 N sodium hydroxide solutions. Because *n*-hexane was used as the washing solvent and could not be injected directly into the LC–MS/MS instrument, the partitioning efficiency of *n*-hexane was calculated indirectly by basifying the remaining aqueous phase and extracting with acetonitrile. As shown in Fig. 4, nereistoxin was quantitatively extracted with acetonitrile at pH 8 or higher but not with *n*-hexane at pH 5 or lower. Hence, the optimal pH values for removing nonpolar interferences and for nereistoxin extraction were equal to 5 and 9, respectively.

The theoretically estimated pK_a_ of nereistoxin was approximately 7 because its dissociated or undissociated form predominated (> 99%) when the difference between the pK_a_ and pH values was greater than 2. Ultimately, the pH-dependent reversible acid–base partitioning and SALLE process were successfully optimized, which allowed for a sufficiently high purification efficiency without the introduction of additional clean-up steps through the simultaneous removal of polar and nonpolar interferences.

### Matrix effect

The enhancement or suppression of an analyte signal during MS ionization can occur due to the coelution of matrix components with the analyte of interest, which represents a so-called ME. If the ME is significant and produces a negative impact on the quality metrics of the analysis procedure (such as accuracy and precision), matrix-matched calibration must be performed to compensate for the ME during MS analysis. To quantitatively determine the ME, nereistoxin calibration curves were constructed for all matrices of the animal-derived foods and solvents in the range of 0.3–20 μg/L, after which their matrix/solvent slope ratios were calculated. If the ME > 0, the analyte signal was considered enhanced; if the ME < 0, it was considered suppressed. The ionization of nereistoxin in the positive ESI mode was enhanced for all extracts of the animal-derived foods except for milk. The obtained nereistoxin MEs ranged from − 6.63% to 9.04% (3.97% for beef, 5.78% for pork, 5.37% for chicken, − 6.63% for milk, and 9.04% for eggs). In contrast, the MEs of other analytical methods have been reported to be − 17 to − 60%^14–16^. Because the MEs were lower than ± 10%, their magnitudes could be ignored during nereistoxin quantitation. These results revealed that the polar matrix constituents were completely removed through the pH-dependent reversible acid−base partitioning with salting-out; as a result, the nereistoxin signal was not affected by the matrix.

### Method validation

**Figure 5:**
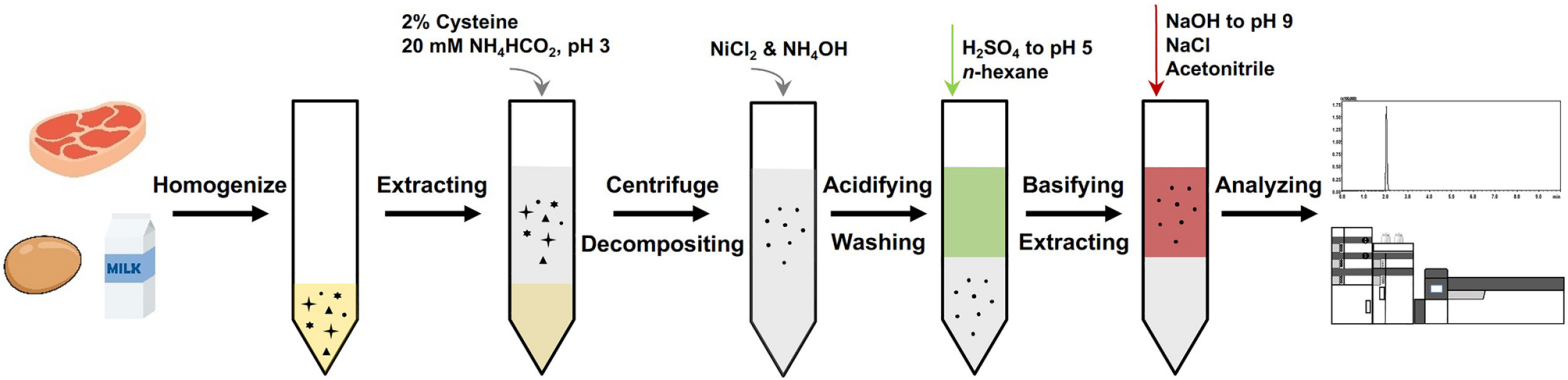
Schematic procedure of the developed method for nereistoxin insecticides in animal-derived foods.

**Figure 6:**
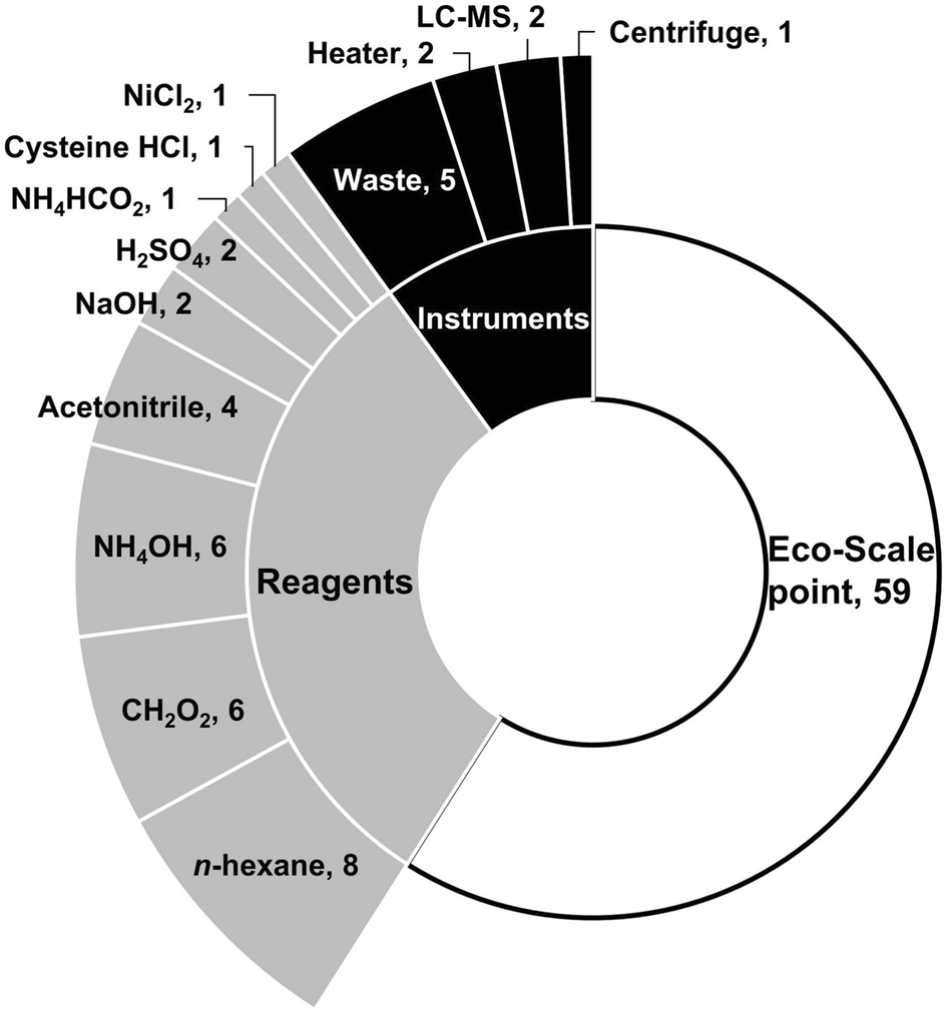
The Eco-Scale points and the penalty points for the developed method.

The residue analysis method developed for the foods of animal origins and optimized in the previous steps was fully validated according to the criteria of the European SANTE/12682/2019 guidelines^41^ (Fig. 5). Selectivity was evaluated by injecting the matrix-matched standard solutions and blank sample extracts. No peak with an S/N ratio greater than three was detected at the nereistoxin retention time, demonstrating that nereistoxin was not subject to any interferences during analysis. The RSD of the retention time was less than 0.6%. The variations in the intensity ratio of the two transition ions were in the range of 5.9–18.6% (8.4% for beef, 5.9% for pork, 9.6% for chicken, 18.6% for milk, and 10.1% for eggs), which were consistent with ± 30% of the average calibration standards, the maximum permitted tolerance for the ion intensity ratio. These results indicate that the detected reside peaks can be easily obtained for real-life samples. The ILOD and ILOQ of nereistoxin were 0.1 and 0.4 μg/L (0.5 and 2.0 pg), respectively, which were 2.5–25 times lower than those of previously reported instrumental analyses^14,16^. The MLOQ of nereistoxin was 2 μg/kg for all foods of animal origins. This value was sufficient to detect trace levels of nereistoxin insecticides and nereistoxin in livestock and poultry products. Considering the nereistoxin conversion factors for NIs, the MLOQs of cartap, bensultap, and thiocyclam were equal to 3.2, 5.8, and 2.4 μg/kg, respectively. The linearity of nereistoxin in the extracts of beef, pork, chicken, milk, and eggs was evaluated by performing linear regression of the matrix-matched calibration curves in the range of 0.4–20 μg/L. Coefficients of determination (R^2^) higher than 0.998 were obtained for nereistoxin in all foods of animal origins, which satisfied the criteria of > 0.98. The RSDs of the peak areas calculated for beef, pork, chicken, milk, and eggs were 0.3%, 0.2%, 0.6%, 0.4%, and 0.1%, respectively. The accuracy and precision of the developed analysis method were assessed by conducting recovery efficiency tests (Fig. 5). According to Table 3, the average recovery efficiencies from various matrices varied between 89.2 and 109.9% with RSDs ranging from 0.9 to 8.9%, which were in good agreement with the average recovery values of 70–120% and RSDs ≤ 20% specified in the European SANTE/12682/2019 guidelines. For the greenness assessment, the developed method had 41 penalty points and 59 Eco-Scale points (Fig. 6) due to the use of various types of reagents in the extraction and decomposition procedures, which was considered an acceptable green method.

**Table 3:** Recovery efficiencies (n = 3) determined for the nereistoxin insecticides and nereistoxin in foods of animal origins.

Pesti -cides	Forti -fication	Recovery, %				
		Beef	Pork	Chicken	Milk	Eggs
Cartap	MLOQ	100.2 ± 5.3	100.2 ± 3.8	104.1 ± 5.1	92.2 ± 4.5	95.9 ± 4.6
	10MLOQ	102.1 ± 3.2	95.1 ± 5.2	102.8 ± 5.0	95.3 ± 4.3	94.0 ± 6.2
	50MLOQ	104.4 ± 3.6	101.6 ± 4.3	100.8 ± 4.5	97.7 ± 2.6	95.5 ± 2.7
Ben -sultap	MLOQ	94.4 ± 4.9	95.1 ± 6.3	92.5 ± 8.5	93.1 ± 1.3	89.2 ± 5.5
	10MLOQ	109.2 ± 6.6	100.2 ± 3.5	109.9 ± 6.8	95.9 ± 4.0	92.8 ± 3.5
	50MLOQ	106.2 ± 2.5	106.3 ± 2.8	95.9 ± 5.5	108.3 ± 3.0	93.6 ± 6.8
Thiocy -clam	MLOQ	100.8 ± 7.2	99.8 ± 2.5	96.4 ± 5.8	93.4 ± 1.9	98.2 ± 1.6
	10MLOQ	94.8 ± 3.8	96.6 ± 6.5	96.4 ± 0.9	97.7 ± 2.5	92.6 ± 2.4
	50MLOQ	94.1 ± 3.9	91.7 ± 1.3	98.1 ± 6.3	95.6 ± 7.8	96.3 ± 3.2
Nereis -toxin	MLOQ	99.8 ± 5.6	98.1 ± 5.4	101.4 ± 4.0	92.4 ± 1.5	93.4 ± 4.6
	10MLOQ	97.4 ± 6.6	93.7 ± 8.9	102.8 ± 4.9	104.2 ± 3.8	105.7 ± 6.0
	50MLOQ	93.7 ± 4.7	99.9 ± 6.9	98.3 ± 4.4	92.0 ± 4.8	92.6 ± 4.6

## Conclusion

The residue analysis method developed in this study was optimized for each step, including extraction, hydrolysis, purification, and instrumental analysis, was validated for all analytical parameters, and it satisfied the criteria of the European SANTE/12682/2019 guidelines. According to the definition of NI residues in foods of plant origins, a common moiety method to quantify nereistoxin, a metabolite of NIs, was developed. For nereistoxin quantitation with LC–MS/MS, three different analytical columns, including C18, HILIC, and Amide columns, were investigated. Among these columns, the Amide column, a HILIC column with a trifunctional amide ligand, exhibited the best chromatographic performance. In previous studies, organic solvents such as acetonitrile and ethyl acetate have been used to extract NIs and nereistoxin from various matrices; however, these solvents were not found to have satisfactory extraction efficiency for all NIs, particularly for bensultap. Therefore, an acidic cysteine solution was selected as the extraction solvent for all NIs and nereistoxin. Additionally, 20 mM ammonium formate at pH 3 was utilized to keep the pH of the extracts at 4–5 for the best extraction efficiency. The NIs in the extracts were decomposed to nereistoxin by alkaline hydrolysis. Since the hydrolysate was based on water, it was difficult to apply d-SPE for purification. DLLME with different kinds of dispersive solvents has been mainly developed for GC analyses. Therefore, pH-dependent acid–base partitioning coupled with SALLE using acetonitrile was performed for purification and for the direct introduction of the extracts into LC–MS/MS. As a result, the matrix effects were less than ± 10%, indicating that this sample preparation procedure has high purification efficiency. The consistent column retention (RSD < 0.6%), high sensitivity (MLOQ 2 μg/kg), high linearity of the calibration curves (R^2^ > 0.998), and excellent instrumental repeatability (RSD < 1%) demonstrated that the LC–MS/MS analysis coupled with the HILIC Amide column separation represented the optimal configuration for high selectivity, sensitivity, accuracy, and precision. Moreover, the developed analytical method exhibited satisfactory precision and accuracy for NIs and nereistoxin in animal-derived food samples according to the SANTE guidelines. The obtained results demonstrated that the proposed method is suitable for the routine analysis and monitoring of trace NIs in foods of animal origins by Korean authorities. Therefore, it can potentially be utilized as an official analytical method for compliance with legislations and for managing the safety of animal commodities.

## Data Availability

The datasets used and analysed during the current study are available from the first author on reasonable request.

## Abbreviations

NIs: Nereistoxin insecticides
MRLs: Maximum residue limits
LLE: Liquid–liquid extraction
SPE: Solid-phase extraction
SPME: Solid-phase microextraction
d-SPE: Dispersive SPE
GC–FPD: Gas chromatography–flame photometric detector
GC–MS: Gas chromatography–mass spectrometry
LC–MS: Liquid chromatography–mass spectrometry
*N*: Theoretical plates
*H*: Height of theoretical plates
ESI: Electrospray ionization
ME: Matrix effects
LODs: Limits of detection
LOQs: Limits of quantitation
RSD: Relative standard deviation
MLOQs: Matrix-dependent method limits of quantitation
HILIC: Hydrophilic interaction liquid chromatography
SALLE: Salting-out-assisted liquid–liquid extraction
